# Interventions to reduce antimicrobial use in livestock: a systematic review and evidence map

**DOI:** 10.2471/BLT.25.294408

**Published:** 2026-03-30

**Authors:** Fiona Emdin, Kayla Strong, Jaskeerat Singh, Daniela Corno, Susan Rogers Van Katwyk, Heather Ganshorn, Arne Ruckert, Jeremy M Grimshaw, Mathieu JP Poirier

**Affiliations:** a Global Strategy Lab, Dahdaleh Institute for Global Health Research, Faculty of Health and Osgoode Hall Law School, York University, Dahdaleh Building 2120, 4700 Keele Street, Toronto, Ontario, M3J 1P3, Canada.; b Libraries and Cultural Resources, University of Calgary, Calgary, Canada.; c Methodological and Implementation Research Program, Ottawa Hospital Research Institute, Ottawa, Canada.

## Abstract

**Objective:**

To identify and describe government policy targeting veterinary antimicrobial use and antimicrobial resistance in production animals globally and to map these interventions to identify evidence gaps.

**Methods:**

We searched MEDLINE, CAB Abstracts, Web of Science and ProQuest Dissertations and Theses Global databases from inception to April 2025 for studies that quantitatively assessed the impact of government-enacted policy interventions by measuring the change in antimicrobial use (for example, mg/kg biomass, mg of active ingredient or defined daily doses for animals) or antimicrobial resistance in production animals or humans. Two reviewers independently completed screening, data extraction and risk-of-bias assessment for identified studies.

**Findings:**

We identified 40 policy evaluations, all originating from the World Health Organization (WHO) Region of the Americas, European Region or Western Pacific Region. No eligible studies from other WHO regions were identified. Most studies (35/40) evaluated legislative or regulatory interventions and 26 were primarily bans or restrictions on specific antimicrobial uses. Most (34/40) articles reported decreases in antimicrobial use in animals or in antimicrobial resistance in animals or humans that were associated with policy implementation. However, the studies were methodologically heterogeneous and had a high risk of bias.

**Conclusion:**

This systematic review and evidence map fill an important gap in knowledge supporting evidence-informed action on antimicrobial resistance. However, we identified relatively few quantitative evaluations of government policies targeting veterinary antimicrobial use or resistance, which highlights the need for additional, methodologically robust, policy evaluations.

## Résumé

**Objectif:**

Identifier et décrire les politiques gouvernementales ciblant l’utilisation d’antimicrobiens à usage vétérinaire et la résistance aux antimicrobiens chez les animaux d’élevage à l’échelle mondiale et cartographier ces interventions afin de mettre en évidence les lacunes dans les données probantes.

**Méthodes:**

Nous avons effectué des recherches dans les bases de données MEDLINE, CAB Abstracts, Web of Science et ProQuest Dissertations and Theses Global, depuis leur création jusqu’en avril 2025, afin de trouver des études évaluant sur le plan quantitatif l’impact des interventions politiques mises en œuvre par différents pouvoirs publics en mesurant l’évolution dans l’utilisation d’antimicrobiens (p. ex. milligrammes par kilogramme de biomasse, milligrammes de principe actif ou doses quotidiennes définies pour les animaux) ou de la résistance aux antimicrobiens chez les animaux d’élevage ou les humains. Deux évaluateurs ont procédé de manière indépendante à la sélection, à l’extraction des données et à l’évaluation du risque de biais pour les études identifiées.

**Résultats:**

Nous avons identifié 40 évaluations de politiques, toutes provenant des régions Amériques, Europe ou Pacifique occidental de l’Organisation mondiale de la Santé (OMS). Aucune étude éligible provenant d’autres régions de l’OMS n’a été identifiée. La plupart des études (35 sur 40) ont évalué des interventions législatives ou réglementaires, et 26 d’entre elles avaient principalement trait à des interdictions ou à des restrictions concernant des utilisations spécifiques d’antimicrobiens. La plupart des articles (34 sur 40) faisaient état d’une diminution de l’utilisation d’antimicrobiens chez les animaux ou de la résistance aux antimicrobiens chez les animaux ou les humains, associée à la mise en œuvre des politiques. Toutefois, les études présentaient une grande hétérogénéité méthodologique et comportaient un risque élevé de biais.

**Conclusion:**

Cette revue systématique et cette cartographie des données probantes comblent une lacune importante dans les connaissances qui étayent des mesures fondées sur des données probantes en matière de résistance aux antimicrobiens. Toutefois, nous avons identifié relativement peu d’évaluations quantitatives des politiques gouvernementales ciblant l’utilisation d’antimicrobiens ou la résistance aux antimicrobiens en médecine vétérinaire, soulignant la nécessité de mener des évaluations supplémentaires et méthodologiquement rigoureuses de ces politiques.

## Resumen

**Objetivo:**

Identificar y describir las políticas gubernamentales dirigidas al uso de antimicrobianos en veterinaria y a la resistencia a los antimicrobianos en animales de producción a nivel mundial, así como mapear estas intervenciones para identificar lagunas de evidencia.

**Métodos:**

Se realizaron búsquedas en las bases de datos MEDLINE, CAB Abstracts, Web of Science y ProQuest Dissertations and Theses Global desde su inicio hasta abril de 2025 para identificar estudios que evaluaran cuantitativamente el impacto de intervenciones de política implementadas por gobiernos, mediante la medición de cambios en el uso de antimicrobianos (por ejemplo, mg/kg de biomasa, mg de principio activo o dosis diarias definidas para animales) o en la resistencia a los antimicrobianos en animales de producción o en humanos. El cribado, la extracción de datos y la evaluación del riesgo de sesgo de los estudios identificados fueron realizados de forma independiente por dos revisores.

**Resultados:**

Se identificaron 40 evaluaciones de políticas, todas procedentes de la Región de las Américas, la Región Europea o la Región del Pacífico Occidental de la Organización Mundial de la Salud (OMS). No se identificaron estudios elegibles procedentes de otras regiones de la OMS. La mayoría de los estudios (35/40) evaluaron intervenciones legislativas o regulatorias, y 26 consistieron principalmente en prohibiciones o restricciones del uso de antimicrobianos específicos. La mayoría de los artículos (34/40) informaron de disminuciones en el uso de antimicrobianos en animales o en la resistencia a los antimicrobianos en animales o en humanos asociadas a la implementación de políticas. No obstante, los estudios presentaron heterogeneidad metodológica y un alto riesgo de sesgo.

**Conclusión:**

Esta revisión sistemática y mapa de evidencia cubren una importante laguna de conocimiento que respalda la adopción de medidas fundamentadas en la evidencia frente a la resistencia a los antimicrobianos. Sin embargo, se identificaron relativamente pocas evaluaciones cuantitativas de políticas gubernamentales dirigidas al uso o la resistencia a los antimicrobianos en veterinaria, lo que pone de manifiesto la necesidad de realizar evaluaciones de políticas adicionales con metodologías sólidas.

## ملخص

**الغرض:**

تحديد ووصف السياسة الحكومية التي تستهدف استخدام مضادات الميكروبات البيطرية، ومقاومة مضادات الميكروبات في حيوانات الإنتاج على مستوى العالم، ورسم خريطة لهذه التدخلات لتحديد الثغرات في الأدلة.

**الطريقة:**

قمنا بالبحث في قواعد بيانات MEDLINE، وCAB Abstracts، وWeb of Science، وProQuest Dissertations، وTheses Global منذ إنشائها وحتى أبريل/نيسان 2025 عن الدراسات التي قامت بالتقييم الكمي لأثر التدخلات السياسية الحكومية، من خلال قياس التغير في استخدام مضادات الميكروبات (على سبيل المثال، ملجم/كجم من الكتلة الحيوية، أو ملجم من المادة الفعالة، أو الجرعات اليومية المحددة للحيوانات) أو مقاومة مضادات الميكروبات في حيوانات الإنتاج أو البشر. قام اثنان من المراجعين بشكل مستقل بإجراء الفحص، واستخراج البيانات، وتقييم مخاطر التحيز للدراسات المحددة.

**النتائج:**

قمنا بتحديد 40 تقييمًا للسياسات، جميعها صادرة عن إقليم الأمريكتين، أو الإقليم الأوروبي، أو إقليم غرب المحيط الهادئ، التابعة لمنظمة الصحة العالمية (WHO). لم يتم العثور على أية دراسات مؤهلة من أقاليم أخرى تابعة لمنظمة الصحة العالمية. قامت معظم الدراسات (35 من 40) بتقييم التدخلات التشريعية أو التنظيمية، بينما ركزت 26 دراسة منها بشكل أساسي على حظر أو تقييد استخدامات محددة لمضادات الميكروبات. أوضحت معظم المقالات (34 من 40) الانخفاضات في استخدام مضادات الميكروبات في الحيوانات، أو في مقاومة مضادات الميكروبات لدى الحيوانات أو البشر، وهو ما ارتبط بتطبيق السياسات. مع ذلك، اتسمت الدراسات بتفاوت منهجياتها، كما أنها عُرضة لخطر التحيز.

**الاستنتاج:**

تعمل هذه المراجعة المنهجية وخريطة الأدلة في سدّ فجوة معرفية هامة تدعم اتخاذ إجراءات قائمة على الأدلة بشأن مقاومة مضادات الميكروبات. ومع ذلك، قمنا بتحديد عدد قليل نسبيًا من التقييمات الكمية للسياسات الحكومية التي تستهدف استخدام مضادات الميكروبات البيطرية أو مقاومتها، مما يركز على الحاجة إلى تقييمات منهجية قوية وإضافية للسياسات.

## 摘要

**目的:**

旨在确定和说明针对全球生产动物体内的兽用抗微生物药物使用量以及抗微生物药物耐药性的政府政策，并梳理此类干预措施以确定证据缺口。

**方法:**

我们搜索了医学文献分析和检索系统在线 (MEDLINE)、CAB Abstracts、Web of Science 和 ProQuest 全球博硕士论文全文数据库自创建起至 2025 年 4 月的相关数据，以查找那些通过测量生产动物或人类体内抗微生物药物使用量【例如：生物量（单位：毫克/千克）、活性成分（单位：毫克）或动物的限定日剂量】或抗微生物药物耐药性变化来定量评估政府所推出政策干预措施所产生影响的研究。基于选定研究，两名评审员独立完成了筛选、数据提取及偏倚风险评估工作。

**结果:**

我们选定的 40 项政策评估研究全部来自于世卫组织 (WHO) 美洲区域、欧洲区域或西太平洋区域。我们在世卫组织的其他区域未找到任何符合条件的研究。大多数研究 (35/40) 的评估对象是立法或监管干预措施，而其中 26 项研究的主要评估对象则是抗微生物药物特定用途相关禁令或限制。据大多数 (34/40) 文章报告，随着政策的落实，动物体内的抗微生物药物使用量有所减少或者动物或人类体内的抗微生物药物耐药性有所降低。然而，此类研究在方法论上具有异质性，且偏倚风险较高。

**结论:**

本系统评价和证据图弥补了知识体系中存在的巨大缺口，从而支持针对抗微生物药物耐药性问题采取循证行动。然而，我们发现针对兽用抗微生物药物使用量或耐药性的政府政策定量评估研究相对较少，这凸显了开展其他在方法论上比较严谨的政策评估研究的必要性。

## Резюме

**Цель:**

Выявить и описать политику правительства, направленную на применение антимикробных препаратов в ветеринарии и на явление устойчивости к антимикробным препаратам у сельскохозяйственных животных, а также систематизировать эти меры для выявления пробелов в доказательной базе.

**Методы:**

Авторы провели поиск по базам данных MEDLINE, CAB Abstracts, Web of Science, ProQuest Dissertations и Theses Global от момента их создания до апреля 2025 года. Целью поиска были исследования, в которых проводилась количественная оценка влияния мер, реализуемых в рамках осуществляемых правительствами политик. Оценка проводилась путем измерения изменений в применении антимикробных препаратов (например, в мг/кг биомассы, мг действующего вещества или в заданных суточных дозах для животных) или путем определения устойчивости к антимикробным препаратам у сельскохозяйственных животных или человека. Два рецензента независимо друг от друга провели отбор, извлечение данных и оценку риска систематической ошибки для выявленных исследований.

**Результаты:**

Авторы обнаружили 40 оценок политики, выполненных в Американском, Европейском и Западно-Тихоокеанском регионах Всемирной организации здравоохранения (ВОЗ). В других регионах ВОЗ не было выявлено соответствующих критериям исследований. Большинство исследований (35/40) были посвящены оценке законодательных или нормативных мер, из них 26 были в основном запретами или ограничениями применения определенных антимикробных препаратов. В большинстве статей (34/40) сообщалось о сокращении интенсивности применения антимикробных препаратов у животных или о снижении устойчивости к антимикробным препаратам у животных и людей, что ассоциировалось с реализацией соответствующей политики. Однако исследования были методологически неоднородными и имели высокий риск систематической ошибки.

**Вывод:**

Этот систематический обзор и карта доказательной базы восполняют существенный пробел в знаниях, необходимых для поддержки действий в отношении устойчивости к антимикробным препаратам на основе доказательной базы. Однако авторы обнаружили относительно мало количественных оценок государственных политик в отношении использования антимикробных препаратов или устойчивости к ним в ветеринарии, что подчеркивает потребность в дополнительной, методологически надежной оценке политик.

## Introduction

In 2020, antimicrobial resistance was estimated to have contributed to almost 5 million human deaths,^1^ making it one of the most critical threats to public health and one that will only continue to grow.^2^ A major driver of antimicrobial resistance is the global use of antimicrobials,^3^ two thirds of which are allotted to production animals,^2^^,^^3^ defined as animals raised to provide a product, such as meat, eggs or milk.^4^ Antimicrobial use in these animals is a driver of antimicrobial resistance in both animal and human populations.^5^^,^^6^

In response to the threat of growing antimicrobial resistance, bodies such as the World Health Organization (WHO), the World Organisation for Animal Health, the United Nations Environment Programme and the Food and Agriculture Organization have called for actions targeting antimicrobial use and resistance in production animal systems and veterinary medicine.^7^ However, to meet this call, governments need evidence on the effectiveness of policy interventions addressing antimicrobial use and resistance in production animals.

To date, reviews of policy on antimicrobial use and resistance in production animals have focused on specific production systems (e.g. feedlots) or specific policy types.^3^ There is a need for research that comprehensively examines government-enacted policies targeting antimicrobial use or resistance across all production animal systems. To fill this knowledge gap, support policy-makers and guide governments, we conducted a systematic review to: (i) identify and describe government policy interventions that have targeted veterinary antimicrobial use and resistance in production animals; and (ii) identify gaps in the evidence supporting these interventions.

## Methods

A veterinarian developed our literature search strategy based on a previously published systematic review and meta-analysis that examined restricting the use of antibiotics in food-producing animals.^3^ An epidemiologist reviewed the search strategy to ensure no key terms were missing. Finally, a veterinary information specialist refined it. The antibiotics included in the search were derived from the World Organisation for Animal Health’s list of antimicrobials important for veterinary medicine.^8^

We searched the databases MEDLINE, CAB Abstracts, Web of Science and ProQuest Dissertations and Theses Global (a grey literature source) from inception to 20 April 2025 for studies quantitatively assessing the impact of a government policy intervention that targeted antimicrobial use or antimicrobial resistance in production animals. No limits on publication dates were applied. We included studies published in any language, and non-English studies were translated using Google Translate, which the Cochrane Handbook for Systematic Reviews of Interventions states is an acceptable method for screening and data extraction when professional translation is not feasible, as are other translation tools.^9^ Finally, we included any identified dissertations or non-peer-reviewed publications.

A list of inclusion and exclusion criteria for title and abstract screening is shown in Table 1. As the objective of this review was to evaluate policies that were enacted or formally endorsed by government authorities, we excluded industry-led initiatives and interventions implemented solely by professional veterinary bodies. Although industry- or profession-led initiatives may be implemented in response to, or in anticipation of, changes in governmental policy, these interventions vary widely in scope, enforcement and accountability, which limits their comparability and their relevance to an evidence synthesis focusing on government policy. At full-text screening, we incorporated one additional criterion: eligible studies had to examine a policy intervention that was enacted by, or implemented in partnership with, a national, state, provincial, regional or municipal government or a government-controlled agency, ministry or department.

**Table 1:** Title and abstract screening criteria, systematic review of government policy interventions to reduce antimicrobial use in production animals, 1979–2022

General criterion	Study inclusion criteria	Study exclusion criteria
**The study assessed the population and disease of interest**	The study reported the impact of a policy intervention targeting production animals, which are defined as any species of animal raised to produce a product for human consumption, including meat, eggs, milk, fur, leather and wool. Included are commonly farmed species, such as avian, bovine, caprine, equine and ovine species, swine, camel, rabbit, fish, bees, molluscs, mink, ferrets and crustaceans, as well as other aquatically farmed species. Less commonly farmed groups, such as other rodents and other aquatic species of animals are also included	The study did not report on a policy intervention that addressed antimicrobial use or resistance in production animals
**The study examined an intervention of interest**	The study assessed the impact of any type of policy intervention on antimicrobial use or resistance in production animals. Policy interventions were defined using the behaviour change wheel framework as those that lead to a change in antimicrobial use through education, persuasion, incentivization, coercion, training, restriction, changing the physical or social context, modelling appropriate behaviour or reducing barriers to action^10^	The intervention was not a policy intervention
**The study quantitatively examined the impact of the intervention**	The study involved quantitative measurement of the impact of the policy intervention. Studies could examine the impact over time, where the comparator group could be historical (i.e. the same population compared before and after an intervention), or could involve a comparable group not subject to the intervention (e.g. with intervention and control sites)	The study did not evaluate the impact of an intervention or was a narrative review, a systematic review or theoretical (e.g. an editorial or commentary)
**The study assessed a primary outcome of interest**	The study reported on an outcome of interest, specifically: (i) the change in antimicrobial use in production animals, including the quantity of antimicrobials administered, prescribed or sold (other measures of antimicrobial use were also acceptable); (ii) the change in antimicrobial resistance in production animals, including the absolute or relative change in phenotypic or genotypic resistance; or (iii) the change in antimicrobial resistance in humans, including the absolute or relative change in phenotypic or genotypic resistance. All routes of antimicrobial administration were eligible (e.g. oral, parenteral and topical)	The study did not report the impact a policy that targeted antimicrobial use or resistance in production animals had on antimicrobial use or resistance in production animals or humans

For studies on antimicrobial use, we included any study that collected data on selling, purchasing, prescribing or administering an antimicrobial drug by veterinarians, livestock producers, insurers or regulatory agencies. Studies that involved measuring antimicrobial consumption could employ any unit of measurement of antimicrobial use, such as sales volume, prescription volume and the amount administered. Examples are: (i) the quantity of active ingredient per unit biomass; (ii) the quantity of active ingredient per population correction unit or estimated live weight of animals; (iii) defined daily dose for animals; (iv) defined course dose; or (v) total sales volume. All routes of antimicrobial administration were eligible for inclusion.

Studies on antimicrobial resistance could involve the measurement of either phenotypic resistance (e.g. the prevalence of bacterial isolates with a breakpoint that indicates resistance) or genotypic resistance (e.g. the prevalence of a bacterium carrying a resistance gene of interest). Studies could also involve the collection of samples from an abattoir, retail unit or farm (e.g. faecal samples). For human studies, data on antimicrobial resistance could be derived from regional or national, routine surveillance databases (e.g. from hospitals) or could have been collected for the study itself. Data on absolute or relative changes in antimicrobial resistance were also included to help interpret the impact of government policy interventions.

### Data analysis

Two reviewers independently screened titles, abstracts and full texts using a systematic review tool (Covidence, Melbourne, Australia). Any disagreements were resolved through consensus or consultation with a third reviewer. Then, two reviewers independently extracted data on the general study characteristics, the policy intervention, the principal impact of the intervention, and any secondary impacts using a tailored Excel extraction form (Microsoft Corporation, Redmond, United States of America). Any conflicts were resolved by consensus. When a consensus could not be reached, a third reviewer made the final decision.

From each included study, we extracted information about: (i) study design; (ii) country and WHO region; (iii) policy category (as defined by the behaviour change wheel framework);^10^ (iv) policy option (e.g. policies banning antimicrobial types in certain production species and policies banning types of antimicrobial use in production animals); and (v) the government level at which the policy was enacted (e.g. national, state, provincial or municipal). As we did not identify three or more studies whose characteristics could be meaningfully pooled, a meta-analysis was not performed.

The risk of bias was assessed across all studies concurrently with the data extraction by two independent reviewers and any disagreements were resolved by consensus. We used the ROBINS-I risk-of-bias assessment tool because it could be applied across the study designs we identified.^11^ The tool is applicable to nonrandomized studies and categorizes them as having a low, moderate, serious or critical risk of bias across seven domains.

We adapted the protocol and methods used in our systematic review from a previously published systematic review and evidence map, which identified and described government policy interventions to reduce human antimicrobial use.^12^ Our review was developed in line with the Preferred Reporting Items for Systematic Reviews and Meta-Analyses guidelines.^13^ The protocol was registered with Open Science Framework (Center for Open Science; https://doi.org/10.17605/OSF.IO/56PZG) and published in the journal *Systematic Reviews*.^4^

## Results

**Fig. 1:**
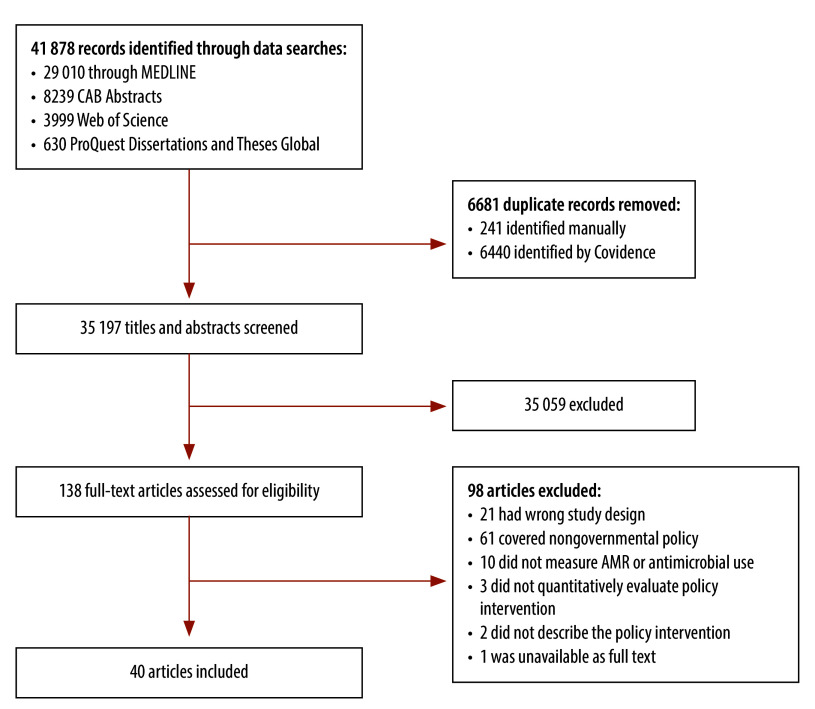
Study selection, systematic review of government policy interventions to reduce antimicrobial use in production animals, 1979–2022

We screened the titles and abstracts of 35 197 citations and we assessed the full texts of publications on 138 studies for eligibility (Fig. 1). In total, 40 studies met the inclusion criteria. Most (37/40) were uncontrolled, before–after studies.^14^^–^^50^ Three were controlled, before–after studies: two had single time-points before and after the intervention,^51^^,^^52^ and one involved an interrupted time series.^53^ Of the 40 studies, 15 evaluated interventions in the WHO European Region,^14^^–^^17^^,^^23^^,^^24^^,^^27^^,^^30^^–^^32^^,^^35^^,^^37^^,^^39^^,^^48^^,^^50^ 12 evaluated interventions in the WHO Region of the Americas,^18^^–^^22^^,^^25^^,^^28^^,^^29^^,^^33^^,^^42^^,^^44^^,^^49^ and 13 evaluated interventions in the Western Pacific Region (Table 2; available at https://www.who.int/publications/journals/bulletin/). ^26^^,^^34^^,^^36^^,^^38^^,^^40^^,^^41^^,^^43^^,^^45^^–^^47^^,^^51^^–^^53^ No studies from the WHO African, Eastern Mediterranean or South-East Asia Regions were identified.

AMR: antimicrobial resistance.

Note: Covidence (Veritas Health Innovation, Melbourne, Australia) is a type of systematic review software.

**Table 2:** Study characteristics, systematic review of government policy interventions to reduce antimicrobial use in production animals, by policy category,^a^ 1979–2022

Study first author and year	Study design	Country (WHO region)^b^	Study period	Data source or biological sample	Policy intervention (year)	Authority enacting policy	Policy option	Outcome measure	Impact of policy	Overall risk of bias^c^
**Guidelines**										
Ungemach et al., 2006^37^	Uncontrolled before–after	Germany (European)	2000–2002	National prescription data	Prudent-use veterinary guidelines (2000)	National government	Antimicrobial guidelines	Animal antimicrobial use	Total veterinary antibiotic consumption declined from 4255 kg pre-guidelines to 1145 kg by the first quarter of 2002	Serious
Sammul et al., 2021^31^	Uncontrolled before–after	Estonia (European)	2010–2019	Wholesaler sales data	National treatment guidelines (2012)	National government	Antimicrobial guidelines	Animal antimicrobial use	Colistin use in pigs declined by 0.23 mg/PUCE per 10 000 *E. coli* vaccine doses over 10 years	Serious
Miguela-Villoldo et al., 2022^27^	Uncontrolled before–after	Spain (European)	1998–2021	Pig caecal samples	Reduce Porcino plan (2016)	National government	Ban on specific antimicrobials	Animal AMR	The prevalence of the colistin resistance-associated *mcr* gene in pig caecal samples increased between 2004 and 2015 to 66% but declined significantly from 2017 (54%) to 2021 (17%; *P* < 0.001)	Serious
Liang et al., 2023 (animals)^52^^,h^	Controlled before–after	China (Western Pacific)	2019–2021	Broiler chicken cloacal swabs	National pilot programme (2018)	National government	Antimicrobial guidelines	Animal AMR	(i) The prevalence of resistance to gentamicin, spectinomycin and ceftiofur was lower in intervention farms compared to non-intervention farms (*P* < 0.05); and (ii) the difference in some other antimicrobial drugs was not significant	Serious
Liang et al., 2023 (antimicrobial resistance genes)^51^^,h^	Controlled before–after	China (Western Pacific)	2019–2021	Broiler chicken cloacal swabs	National pilot programme (2018)	National government	Antimicrobial guidelines	Animal AMR	Abundance of resistance genes declined in intervention farms compared to non-intervention farms (*P* < 0.05)	Serious
Perrin-Guyomard et al., 2023^30^	Uncontrolled before–after	France (European)	2014–2020	Poultry surveillance samples	Ecoantibio2 plan (2017)	National government	Antimicrobial guidelines	Animal AMR	The prevalence of colistin resistance in *E. coli* in poultry populations declined from 6% to 3% in turkeys and from 1.2% to 1.0% in chickens	Serious
**Legislation and regulation**										
Trolldenier et al., 1991^50^	Uncontrolled before–after	Germany (European)	1979–1988	Human clinical isolates and porcine and bovine samples	Ban on oxytetracycline feed use (1983)	State government	Ban on specific antimicrobial uses (e.g. growth promotion)	Animal and human AMR	(i) The prevalence of oxytetracycline resistance declined across Enterobacteriaceae in animals and humans; (ii) the prevalence of oxytetracycline resistance in porcine and bovine *Escherichia coli* decreased by 27% and 17%, respectively, from 1980 to 1988; (iii) the prevalence of oxytetracycline resistance decreased by 65% in bovine *Salmonella typhimurium*, by 50% in porcine *S. typhimurium*, by 50% in bovine *Salmonella dublin* and by 13–30% in porcine *Salmonella choleraesuis*; and (iv) in humans, the prevalence of oxytetracycline resistance in *E. coli* and *Klebsiella* decreased from 50–70% to 30%	Critical
Laine et al., 2004^48^	Uncontrolled before–after	Finland (European)	1999–2000	Farm-level antimicrobial use data	European Union ban on carbadox and olaquindox (1999)	Supranational body	Ban on specific antimicrobial uses (e.g. growth promotion)	Animal antimicrobial use	The distribution of antimicrobial use intensity across farms shifted to higher numbers of low and moderate users of antibiotics, without a change in piglet diarrhoea treatment frequency	Serious
Bywater et al., 2005^16^	Uncontrolled before–after	European Union (European)	1998–2000	Chicken and pig slaughter and faecal samples	European Union ban on four growth promoters (1999)	Supranational body	Ban on specific antimicrobials	Animal AMR	The prevalence of resistance in *E. faecium* declined for spiramycin, tylosin and virginiamycin but not bacitracin, across the six countries examined	Critical
Lauderdale et al., 2007^26^	Uncontrolled before–after	China, Taiwan (Western Pacific)	2000–2003	Broiler chicken faecal samples	Avoparcin ban (2000)	National government	Ban on specific antimicrobial uses (e.g. growth promotion)	Animal AMR	The prevalence of vancomycin-resistant *Enterococcus* spp. declined in broiler chicken: from 13.7% to 3.7% for *E. faecalis* and from 3.4% to 0% for *E. faecium*	Serious
Kmet’ et al., 2007^24^	Uncontrolled before–after	Slovakia (European)	2005–2007	Broiler chicken faecal samples	European Union in-feed antibiotic ban (2007)	Supranational body	Ban on specific antimicrobial uses (e.g. growth promotion)	Animal AMR	The prevalence of resistance of *E. coli* to enrofloxacin declined from 52% to 38% and resistance to fluoroquinolones declined from 54% to 33%, with minor increases in ampicillin and sulbactam resistance (from 0% to 2.7%) noted after the ban	Critical
Aarestrup et al., 2010^14^	Uncontrolled before–after	Denmark (European)	1995–2000	Broiler chicken and pig slaughter samples	European Union avoparcin ban (1995), virginiamycin ban (1998) and voluntary antibiotic growth promoter ban (1999)	National government	Ban on specific antimicrobial uses (e.g. growth promotion)	Animal AMR	The prevalence of glycopeptide-resistant *Enterococcus faecium* and *Enterococcus faecalis* declined markedly in broilers (5.8–72.7%) and pigs (6.0–90%) after growth promoter withdrawal	Serious
Andreasen et al., 2012^15^	Uncontrolled before–after	Denmark (European)	2008–2011	National antimicrobial use data	Yellow card scheme and voluntary cephalosporin ban (2010)	National government	Ban on specific antimicrobial uses (e.g. growth promotion)	Animal antimicrobial use	Total antimicrobial consumption in swine declined by 12.5% between 2009 and 2010 and by 24.5% between 2010 and 2011, with near elimination of cephalosporin use	Serious
Zawack et al., 2016^42^	Uncontrolled before–after	USA (Americas)	2004–2012 (2002–2007 for enrofloxacin analysis)	National Antimicrobial Resistance Monitoring System data	Enrofloxacin withdrawal in poultry (2005)	National government	Ban on specific antimicrobial uses (e.g. growth promotion)	Animal AMR	The prevalence of fluoroquinolone resistance in *Campylobacter* spp. remained largely unchanged following withdrawal of enrofloxacin	Moderate
Callens et al., 2018^17^	Uncontrolled before–after	Belgium (European)	2011–2015	National veterinary antimicrobial use and laboratory AMR data	National reduction targets (2011)	National government	Reduction goals	Animal antimicrobial use and AMR	(i) Antimicrobial use declined across all classes of antimicrobials except phenicols; and (ii) the prevalence of resistance in commensal *E. coli* declined for most antibiotics, with positive antimicrobial use and AMR correlations for ampicillin (*P*: 0.01) ciprofloxacin, nalidixic acid, sulfamethoxazole, tetracycline and trimethoprim (*P*: 0.05)	Serious
Vanhoudt et al., 2018^39^^,d^	Uncontrolled before–after	Netherlands (European)	2013–2015	Farm veterinary records	Ban on preventive antimicrobial use (2013)	National government	Ban on specific antimicrobial uses (e.g. growth promotion)	Animal antimicrobial use	(i) Intramammary antimicrobial sales declined by 38% (dry cow therapy) and 19% (mastitis treatment); (ii) overall use of antimicrobials decreased by 29% between 2013 and 2015; and (iii) the selection of cows for dry cow therapy without antimicrobials did not impact udder health	Serious
Dillon 2020^22^	Uncontrolled before–after	USA (Americas)	2009–2018	National Antimicrobial Resistance Monitoring System and national antimicrobial use databases	Veterinary Feed Directive (2017)	National government	Ban on specific antimicrobial uses (e.g. growth promotion)	Animal and human antimicrobial use and AMR	Significant reductions in the prevalence of: (i) tetracycline resistance in *Salmonella* (cattle, *P*: 0.007; swine, *P*: 0.023; chicken, *P*: 0.007); (ii) penicillin resistance in *Salmonella* (turkey, *P*: 0.009); (iii) penicillin resistance in *Campylobacter* in chickens (*P* < 0.001); and (iv) penicillin resistance in *Campylobacter* in humans (*P*: 0.02), which was in line with reduced antimicrobial use in poultry	Serious
Shen et al., 2020^34^^,e^	Uncontrolled before–after	China (Western Pacific)	2016–2018	Animal, human, food and environmental samples	Ban of colistin in feed (2017)	National government	Ban on specific antimicrobial uses (e.g. growth promotion)	Animal and human AMR	(i) Ecosystem-wide decline in the prevalence of *mcr* genes observed; and (ii) the prevalence of colistin resistance declined from 45% to 19% in pigs (*P* < 0.0001) and from 19% to 5% in humans (*P* < 0.0001)	Serious
Singer et al., 2020^49^^,f^	Uncontrolled before–after	USA (Americas)	2013–2017	Producer-reported antimicrobial use data	Veterinary Feed Directive (2017)	National government	Ban on specific antimicrobial uses (e.g. growth promotion)	Animal antimicrobial use	Between 2013 and 2017: (i) medically important in-feed antimicrobial use declined substantially from 93% to 17%; (ii) in-feed tetracycline use decreased approximately 95%; (iii) water-soluble penicillin use decreased approximately 21%; (iv) water-soluble tetracycline use decreased approximately 47%; and (v) water-soluble lincomycin use decreased approximately 28%	Serious
Wang et al., 2020^40^^,e^	Uncontrolled before–after	China (Western Pacific)	2015–2018	National sales and surveillance data	Ban of colistin in feed (2017)	National government	Ban on specific antimicrobial uses (e.g. growth promotion)	Animal and human antimicrobial use and AMR	(i) Colistin production, sales and resistance declined markedly for animals and humans after the ban on colistin; (ii) after the ban on colistin as a growth promoter, marked reductions were observed in the production (27 170 tonnes in 2015 versus 2 497 tonnes in 2018) and sale (US$ 71.5 million in 2015 versus US$ 8.0 million in 2018) of colistin sulfate; (iii) the prevalence of colistin resistance decreased from 34% to 5.1% in pig faeces (*P* < 0.0001) and from 18.1% to 5% in chicken faeces (*P* < 0.0001) from 2015 to 2018; and (iv) the prevalence of human carriage of colistin-resistant bacteria decreased from 14.3% to 6.3% (*P* < 0.0001) from 2016 to 2019	Moderate
Santman-Berends et al., 2021^32^^,d^	Uncontrolled before–after	Netherlands (European)	2013–2017	National surveillance	Preventive use ban (2013)	National government	Ban on specific antimicrobial uses (e.g. growth promotion)	Animal antimicrobial use	Antimicrobial use declined by 63% in dry cow therapy and by 15% in intramammary applications	Serious
Shen et al., 2021^53^^,e^	Controlled before–after (interrupted time series)	China (Western Pacific)	2011–2019	Human *E. coli* isolates from hospitalized patients	Ban of colistin in feed (2017)	National government	Ban on specific antimicrobial uses (e.g. growth promotion)	Human AMR	(i) The prevalence of colistin resistance genes, which had increased to > 60% by 2016, declined rapidly after the 2017 ban to 5.3%, which approached pre-2015 levels; but (ii) background prevalence remained higher than early baseline levels in 2011–2013	Serious
Tenhagen et al., 2021^35^	Uncontrolled before–after	Germany (European)	2010–2016	Slaughter samples	National AMR minimization strategy (2013)	National government	Antimicrobial guidelines and national strategy	Animal antimicrobial use and AMR	(i) Large reductions in macrolide (−62%) and tetracycline (−58%) use; (ii) smaller reductions in aminoglycoside (−27%) and fluoroquinolone (−15%) use; (iii) an association was observed between reduced antibiotic use and the prevalence of resistance in *Campylobacter* isolates to tetracycline and erythromycin but not to aminoglycosides; and (iv) the prevalence of resistance to nalidixic acid and ciprofloxacin increased despite a decrease of fluoroquinolone use	Moderate
Tu et al., 2021^36^^,e^	Uncontrolled before–after	China (Western Pacific)	2017–2018	Pig faecal swabs	Ban of colistin in feed (2017)	National government	Ban on specific antimicrobial uses (e.g. growth promotion)	Animal AMR	The prevalence of *mcr-1* in pig faeces declined from 86.4% to 5.6% (*P* < 0.01) but the colistin ban had little influence on unrelated resistance genes	Serious
Usui et al., 2021^38^	Uncontrolled before–after	Japan (Western Pacific)	2017–2019	Pig faecal samples	Ban of colistin in feed(2018)	National government	Ban on specific antimicrobial uses (e.g. growth promotion)	Animal AMR	The colistin-resistant *E. coli* concentration declined from 10^7^ to 10^3^–10^4^ CFU/g (*P* < 0.01)	Moderate
de Lagarde Laroche et al., 2022^20^^,g^	Uncontrolled before–after	Canada (Americas)	2017–2020	Farm faecal samples	Restriction of highest-priority antimicrobials (2019)	Provincial government	Restricted use	Animal AMR	The prevalence of multidrug resistance in *E. coli* declined from 83% to 71% (*P*: 0.05)	Serious
Guérin et al., 2022^23^	Uncontrolled before–after	Belgium (European)	2014–2015 and 2020–2021	Calf faecal and necropsy samples	Royal decree restricting cephalosporins (2016)	National government	Ban on specific antimicrobials	Animal AMR	(i) The prevalence of resistance to 3rd and 4th generation cephalosporins in *E. coli* declined from 16% to 7.5%; (ii) the prevalence of AmpC 𝛽-lactamases declined from 8% to 1%; and (iii) resistance varied, such that there was an increase in the prevalence of resistance to narrow-spectrum *β*-lactamases	Serious
Laroche et al., 2022^25^^,g^	Uncontrolled before–after	Canada (Americas)	2016–2019	Provincial sales data	Restriction of highest-priority antimicrobials (2019)	Provincial government	Ban on specific antimicrobial uses (e.g. growth promotion)	Animal antimicrobial use	The sale of category-1 antimicrobials for use in dairy cattle declined by 76% from 1.7 to 0.4 mg/ population correction unit	Serious
Millar et al., 2022^28^^,g^	Uncontrolled before–after	Canada (Americas)	2017–2021	Sales and farm data	Restriction of highest-priority antimicrobials (2019)	Provincial government	Ban on specific antimicrobials	Animal antimicrobial use	The sale of category-1 antimicrobials for dairy herds declined substantially from a range of 14 258–21 528 defined course doses per month to 1494–4707 doses per month by 2021	Serious
Wen et al., 2022^46^	Uncontrolled before–after	China (Western Pacific)	2018–2021	*Campylobacter coli* isolates	Antibiotic growth promoter ban (2020)	National government	Ban on specific antimicrobial uses (e.g. growth promotion)	Animal AMR	(i) The prevalence of erythromycin resistance declined from 92% in 2018 to 62% in 2021; but (ii) the prevalence of gentamicin resistance increased from 78% in 2018 to 94% in 2021 and the prevalence of florfenicol resistance increased from 14% in 2018 to 72% in 2021	Serious
Zhang et al., 2022^43^^,e^	Uncontrolled before–after	China (Western Pacific)	2014–2019	Chicken cloacal swabs	Ban of colistin in feed (2017)	National government	Ban on specific antimicrobial uses (e.g. growth promotion)	Animal AMR	The *mcr-1*-positivity rate peaked before the colistin ban, at 12.6% in 2016 and 11.4% in 2017, and then decreased significantly to 1.7% in 2018 and 0.9% in 2019	Serious
Zhao et al., 2022^45^	Uncontrolled before–after	China (Western Pacific)	2009–2019	Human clinical isolates	Ban of colistin in feed (2017)	National government	Ban on specific antimicrobial uses (e.g. growth promotion)	Human AMR	The prevalence of *mcr-1*-positive *E. coli* showed an increasing trend between 2009 and 2016, whereas a decreasing trend was observed after 2017 when colistin was banned as a feed additive	Serious
Casey et al., 2023^18^	Uncontrolled before–after	USA (Americas)	2013–2021	Human urinary *E. coli* isolates	California Senate Bill 27 restricting medically important antibiotics to prescription-only use (2018)	State government	Restricted use	Human AMR	(i) The prevalence of extended-spectrum cephalosporin resistance in *E. coli* declined by 7.1% (*P* < 0.01); but (ii) no significant changes were observed for aminoglycosides, fluoroquinolones or tetracyclines	Moderate
Chandra Deb et al., 2023^19^	Uncontrolled before–after	USA (Americas)	2012–2019	National Antimicrobial Resistance Monitoring System and CDC isolates	Food and Drug Administration stewardship regulations (2015)	National government	Ban on specific antimicrobial uses (e.g. growth promotion)	Animal and human AMR	Across the multiple bacterial species examined: (i) there were minimal phenotypic resistance changes (< 1-fold dilution); and (ii) no consistent reductions in the prevalence of resistance genes	Serious
Hou et al., 2023^47^	Uncontrolled before–after	China (Western Pacific)	2018–2022	Pig manure isolates	Antibiotic growth promoter ban (2020)	National government	Ban on specific antimicrobial uses (e.g. growth promotion)	Animal AMR	(i) The prevalence of multidrug resistance in isolates from pig manure declined; and (ii) the number of antibiotics for which the prevalence of resistance was > 66.7% decreased from six in 2018 to four in Hebei province and to two in Ningxia province in 2022	Serious
Sarkar & Okafor, 2023^33^^,f^	Uncontrolled before–after	USA (Americas)	2002–2019	National Antimicrobial Resistance Monitoring System data	Veterinary Feed Directive (2017)	National government	Restricted use	Animal AMR	(i) The odds of detecting tetracycline-resistant *Escherichia* (odds ratio, OR: 0.60), tetracycline-resistant *Campylobacter* (OR: 0.89) or erythromycin-resistant *Campylobacter* (OR: 0.43) in chicken breast decreased significantly after the Veterinary Feed Directive was issued; (ii) the odds of detecting tetracycline-resistant *Salmonella* (OR: 0.66), *Escherichia* (OR: 0.56) or *Campylobacter* (OR: 0.33) in ground turkey also decreased significantly; but (iii) the odds of detecting tetracycline-resistant *Salmonella* in chicken breast (OR: 1.49) and erythromycin-resistant *Campylobacter* in ground turkey (OR: 4.63) increased significantly	Serious
Yang et al., 2023^41^^,e^	Uncontrolled before–after	China (Western Pacific)	2011–2020	Foodborne surveillance data	Ban of colistin in feed (2017)	National government	Ban on specific antimicrobial uses (e.g. growth promotion)	Human AMR	The prevalence of *mcr* genes in *S. typhimurium* declined from 11.35% in 2017 to 4.31% in 2018 (*P* < 0.0001) and to 0.79% in 2019 (*P* < 0.0001)	Serious
Mosaddegh et al., 2024^29^	Uncontrolled before–after	USA (Americas)	2006–2014	National Antimicrobial Resistance Monitoring System data	Extra-label cephalosporin ban (2012)	National government	Ban on specific antimicrobial uses (e.g. growth promotion)	Animal AMR	The prevalence of multidrug resistance in *S. dublin* declined from 92.41% before 2012 to 88.23% after 2012	Critical
de Lagarde et al., 2024^21^^,g^	Uncontrolled before–after	Canada (Americas)	2017–2021	Farm faecal samples	Restriction of highest-priority antimicrobials (2019)	Provincial government	Ban on specific antimicrobials	Animal AMR	The prevalence of resistant and virulent *E. coli* lineages remained unchanged over a 4-year period on farms despite reduced access to antimicrobials	Serious
Cobo-Angel et al., 2025^44^	Uncontrolled before–after	USA (Americas)	2012–2022	National Antimicrobial Resistance Monitoring System animal samples (slaughter and retail)	Food and Drug Administration cephalosporin restriction (2012) and Veterinary Feed Directive (2017)	National government	Ban on specific antimicrobial uses (e.g. growth promotion)	Animal AMR	The median minimum inhibitory concentration for most antimicrobials in bovine *Salmonella* declined by 10–20%, except for chloramphenicol and gentamicin, which showed increases in median minimum inhibitory concentration over the periods 2013–2017 and 2018–2022	Moderate

AMR: antimicrobial resistance; CDC: Centers for Disease Control and Prevention; CFU: colony forming unit; PUCE: population unit correction for exposure; WHO: World Health Organization; US$: United States dollar.

^a^ Policy categories were defined using the behaviour change wheel framework.^10^

^b^ The full names of the World Health Organization regions listed are the Region of the Americas, the European Region and the Western Pacific Region.

^c^ The risk of bias was assessed using the ROBINS-I tool, which categorizes risk as low, moderate, serious or critical.^11^

^d^ This study examined the impact of the 2013 ban on the preventative use of antimicrobials in Dutch livestock.

^e^ This study examined the impact of a 2017 policy in China that banned colistin as a feed additive.

^f^ This study examined the impact of the 2017 rule changes to the United States Food and Drug Administration’s Veterinary Feed Directive, which aimed to limit the use of medically important antimicrobials in food-producing animals, including for growth promotion.

^g^ This study examined the impact of a 2019 provincial regulation that restricted the use of antimicrobials of very high importance for human health in production animals in Quebec, Canada.

^h^ This study examined the impact of a 2018 Chinese national pilot programme to reduce the use of veterinary antimicrobial drugs by guiding farms towards the rational use of antimicrobial drugs and improved surveillance of bacterial resistance of animal origin.

**Fig. 2:**
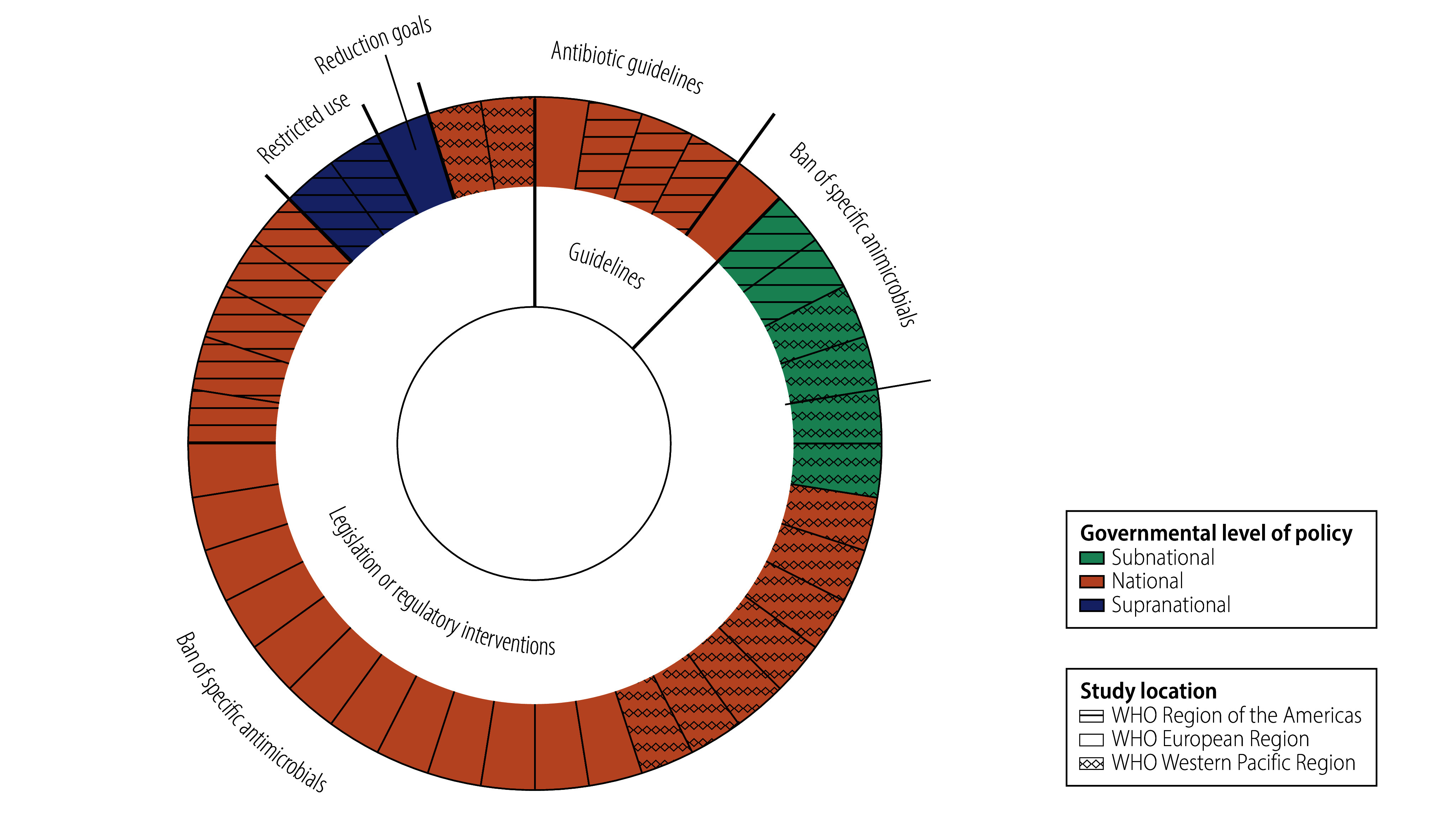
Evidence map of the relationships between the policy category, option and governmental level and study location, systematic review of government policy interventions to reduce antimicrobial use in production animals, 1979–2022

Twenty-six studies evaluated bans on the use of specific antimicrobials, most commonly those targeting growth promotion.^14^^,^^15^^,^^19^^,^^22^^,^^24^^–^^26^^,^^29^^,^^32^^–^^34^^,^^36^^,^^38^^–^^50^^,^^53^ Six studies assessed guideline-based interventions,^30^^,^^31^^,^^35^^,^^37^^,^^51^^,^^52^ and four examined bans on specific classes of antimicrobial.^16^^,^^23^^,^^27^^,^^28^ Three studies evaluated policies restricting antimicrobial use in defined contexts,^18^^,^^20^^,^^21^ and one assessed a policy establishing a target for a reduction in use.^17^ Most policies (35/40) were applied broadly across different species of production animal; a smaller number specifically targeted cows,^32^^,^^39^ chickens^42^^,^^47^ or pigs.^15^ Most policies (31/40) were implemented at a national level,^14^^,^^15^^,^^17^^,^^19^^,^^22^^,^^23^^,^^26^^,^^27^^,^^29^^–^^47^^,^^49^^,^^51^^–^^53^ whereas a few were applied at a state or provincial (i.e. subnational) level,^18^^,^^20^^,^^21^^,^^25^^,^^28^^,^^50^ or at a supranational level (e.g. the European Union).^16^^,^^24^^,^^48^ Seven studies captured the impact of policies on more than one outcome.^17^^,^^19^^,^^22^^,^^34^^,^^35^^,^^40^^,^^50^ The detailed characteristics of each study are listed in Table 2 and the interrelationships between the government level at which policy was determined, the policy category and option, and the WHO region where the study was conducted are illustrated in Fig. 2.

WHO: World Health Organization.

Notes: Each slice in the circle represents an included study. Policy categories are shown in the inner ring and policy options are shown in the outer ring.

### Antimicrobial use in animals

Fourteen studies investigated the impact of policies on antimicrobial use in production animals.^15^^,^^17^^,^^22^^,^^25^^,^^28^^,^^31^^,^^32^^,^^35^^,^^37^^–^^40^^,^^48^^,^^49^ All 14 reported a reduction in antimicrobial use that was attributable to the policies they examined. Reductions ranged from moderate (i.e. around 20% to 40%) to the near-complete elimination of specific classes of antimicrobials. For example, two studies found that the consumption of category-1 antimicrobials, that is, antimicrobials of very high importance for human health, was significantly reduced after a Canadian provincial regulation restricted usage of these antimicrobials in production animals in Quebec in 2019.^25^^,^^28^ One of these two studies found that sales of category-1 antimicrobials declined by 70% to 80% across production sectors following implementation of the provincial legislation,^25^ whereas the other documented a 76% reduction in dairy cattle.^28^ In addition, two studies from Denmark and Kingdom of the Netherlands reported that decreased antimicrobial use was not associated with adverse animal health outcomes.^14^^,^^39^ For example, the Dutch study showed that a 2013 ban on the preventive use of antimicrobials in livestock, which included a ban on the blanket application of antimicrobial dry cow treatment, led to a 63% reduction in this treatment and did not impact udder health.^39^ In Denmark, successful antimicrobial use regulations were supported by enforcement mechanisms, such as monitoring systems and financial penalties for noncompliance.^14^^,^^15^

### Antimicrobial resistance in animals

Twenty-eight studies investigated the impact of policies on antimicrobial resistance in animals.^14^^,^^16^^,^^17^^,^^19^^–^^24^^,^^26^^,^^27^^,^^29^^,^^30^^,^^33^^–^^36^^,^^38^^,^^40^^,^^42^^–^^47^^,^^50^^–^^52^ Fourteen of the 28 reported reductions in antimicrobial resistance following policy implementation. For example, after implementation of a European ban on avoparcin, glycopeptide resistance in *Enterococcus faecium* in Danish pigs declined from 70% to 90% to below 10%.^14^ Similarly, three studies that evaluated China’s 2017 ban on colistin in animal feed reported reductions in the prevalence of the colistin-resistance gene in bacteria of between 40 and 80 percentage points, with the prevalence in some pig populations declining from over 80% before the ban to under 10% after.^34^^,^^36^^,^^40^

However, the effects of policies were not uniform and varied by both pathogen and antimicrobial class. Eleven studies found that the prevalence of resistance declined for some bacterial species or antimicrobials but remained unchanged for others.^16^^–^^18^^,^^23^^,^^24^^,^^35^^,^^36^^,^^44^^,^^46^^,^^47^^,^^52^ One study reported that, although the prevalence of erythromycin resistance decreased among *Campylobacter* isolates from pigs, the prevalence of antibiotic resistance to gentamicin and florfenicol increased between 2018 and 2021 following the 2020 Chinese ban on antibiotic growth promoters in feed.^46^ In addition, three studies did not find any change in the prevalence of antimicrobial resistance caused by the interventions.^19^^,^^21^^,^^42^ One of these studies, which looked at the impact of national drug regulations in the United States, suggested that the ability to detect changes in prevalence may have been reduced by the limited follow-up time after policy implementation. On the other hand, another study documented the persistence of resistance genes despite sustained restrictions.^19^ One study from Quebec, Canada, which investigated the long-term impact of the 2019 provincial policy restricting category-1 antimicrobials, found that, although the prevalence of multidrug-resistance in *Escherichia coli* declined modestly from 83% to 71% over 4 years, resistance genes continued to spread and persist on dairy farms despite the restrictions.^21^

### Antimicrobial resistance in humans

Nine studies evaluated how antimicrobial resistance in humans was affected by policies aimed at reducing antimicrobial use or resistance in animals.^18^^,^^19^^,^^22^^,^^34^^,^^40^^,^^41^^,^^45^^,^^50^^,^^53^ Six studies, one from Germany and five from China,^34^^,^^40^^,^^41^^,^^45^^,^^50^^,^^53^ examined the effect of bans on oxytetracycline and colistin in animal feed. All found an associated reduction in the prevalence of resistance among the human pathogens examined. For example, following China’s colistin feed ban, there were documented declines in the prevalence of a gene associated with colistin resistance in bacteria of 60% to 80%, with the prevalence of resistance falling from 15% to 20% before the feed ban was implemented to under 5% after.^34^^,^^40^^,^^41^^,^^45^^,^^53^ In Germany, restrictions on oxytetracycline use were associated with reductions in the prevalence of oxytetracycline resistance among human Enterobacteriaceae of 15 to 30 percentage points.^50^

A 2023 study that examined the impact of a 2018 Senate bill restricting the use of some antibiotics in the American state of California found it was associated with a 7.1% reduction in the prevalence of extended-spectrum cephalosporin resistance; however, no change was found in resistance to aminoglycosides, fluoroquinolones or tetracyclines.^18^ Similarly, a 2023 study of the effect of a 2015 regulation from the United States Food and Drug Administration to control the use of medically important antimicrobials by requiring veterinary oversight and eliminating their use for growth promotion failed to find any change in the prevalence of resistance genes across human and animal bacterial samples.^19^

### Risk of bias

The overall risk of bias for each of the 40 studies is listed in Table 2. Four studies (10%) were classified as having a critical risk of bias.^16^^,^^24^^,^^29^^,^^50^ Thirty (75%) had a serious risk of bias, predominantly due to confounding variables, potential biases in participant selection and unclear intervention classifications.^14^^,^^15^^,^^17^^,^^19^^–^^23^^,^^25^^–^^28^^,^^30^^–^^34^^,^^36^^,^^37^^,^^39^^,^^41^^,^^43^^,^^45^^–^^49^^,^^51^^–^^53^ Finally, six studies (15%) were classified as having a moderate risk of bias.^18^^,^^35^^,^^38^^,^^40^^,^^42^^,^^44^

## Discussion

Most evaluations of government policies targeting veterinary antimicrobial use that we identified were observational before–after studies conducted in high-income countries in the WHO Region of the Americas, the WHO European Region or the WHO Western Pacific Region. Only a minority examined the impact of policies on antimicrobial resistance in humans. The most frequently evaluated policy categories were legislative and regulatory interventions, and most interventions involved bans on specific antimicrobial classes (e.g. fluoroquinolones) or specific uses of antimicrobials (e.g. for growth promotion or in-feed use) or were guideline-based approaches intended to improve antimicrobial use in production animal systems.

Most studies reported improvements in the outcome of interest due to the various policies evaluated: there were decreases in antimicrobial use, animal antimicrobial resistance or human antimicrobial resistance. However, three studies reported no change in animal antimicrobial resistance after policy implementation,^19^^,^^21^^,^^42^ whereas eleven studies reported reductions in the prevalence of antimicrobial resistance for some pathogens or antimicrobial classes but no change for others.^16^^–^^18^^,^^23^^,^^24^^,^^35^^,^^36^^,^^44^^,^^46^^,^^47^^,^^52^

Fluoroquinolone-resistant microbial strains or resistance genes were reported to persist after policy implementation in three studies, suggesting that a reduction in antimicrobial use may not translate uniformly into reductions in resistance across all pathogen–drug combinations.^18^^,^^35^^,^^42^ These findings align with previous evidence that fluoroquinolone-resistant strains may have greater biological fitness than susceptible strains, which enables them to persist despite interventions addressing fluoroquinolone use.^54^ It is possible, then, that reductions in antimicrobial use alone may be insufficient to eliminate certain resistance phenotypes. Similarly, one study found that the prevalence of resistance to vancomycin and erythromycin in pigs in Denmark did not decrease until the use of both avoparcin and tylosin was restricted,^14^ which highlights the importance of addressing multiple selective pressures simultaneously. Moreover, reductions in antimicrobial use may not result in immediate or measurable declines in resistance because of factors such as the low fitness cost associated with certain resistance traits, the presence of other selective pressures (e.g. co-selection), or the long time lag before changes are observed. Longer follow-up periods are needed to detect the impact of policies on changes in antimicrobial resistance compared with changes in antimicrobial use, especially when assessing downstream effects in humans.^3^

Since most studies included in our analysis were conducted in high-income countries, the generalizability of our findings to different contexts, especially resource-constrained settings, may be limited. For example, Denmark’s yellow card system was more effective when antimicrobial use regulations were accompanied by enforcement mechanisms, including clear sanctions for noncompliance.^15^ In various settings, many antimicrobials are sold without prescriptions by legal drug sellers or on the black market, which may be hard to regulate effectively. Similarly, access to veterinary care and other animal services varies widely internationally,^55^ which further limits the transferability of our findings on policy impacts across regions.

Despite the grave threat posed by antimicrobial resistance,^1^ it is notable that we were able to identify only a relatively small number of evaluations of policy targeting antimicrobial use or resistance in animal systems. A 2019 systematic review and meta-analysis of government policy interventions targeting antimicrobial use in humans found that regulatory and guideline-based interventions were the most commonly evaluated policy types.^12^ We identified far fewer studies in animals compared with the 2019 human health-focused review. This discrepancy may reflect either the less frequent implementation of policies in veterinary contexts or a lack of formal quantitative evaluation of existing policies. Countries that have conducted internal evaluations of veterinary antimicrobial polices should be encouraged to make their findings publicly available to promote best practice for policies and adaptation to other contexts. Researchers should also prioritize the identification and reporting of contextual determinants, such as regulatory capacity, resource constraints and institutional structures, that influence the effectiveness of policy across One Health sectors.

Our study has several limitations. First, like most reviews, publication bias may have affected our results. Although we designed our search strategy to cover multiple data sources, relatively few studies were identified and there were none from the WHO African, Eastern Mediterranean or South-East Asia Regions. Most studies we identified reported that the policies evaluated had a positive impact. Although this may be accurate, it may also reflect a positive publication bias. Moreover, the high risk of bias we identified across most studies also reduces confidence in our findings. Additionally, as many studies focused on time frames close to the intervention, the long-term or permanent impacts of interventions remain unknown. We excluded industry-led interventions, which may have led us to underestimate the total level of antimicrobial stewardship activity in production animal systems, particularly in settings where voluntary or profession-led initiatives preceded or complemented formal regulation. However, this approach was necessary to ensure the focus of our review remained on informing government decision-makers.

Most studies we identified employed uncontrolled, before–after comparisons and most had a high risk of bias. Consequently, there is a need for improved standardized measures for use in intervention research on antimicrobial resistance in production animals. Studies that examined antimicrobial resistance in animals obtained samples for evaluating resistance from a variety of sources, including healthy animals on farms, sick animals at necropsy and retail meat. This variability reduced the generalizability of our findings. In addition, there were variations in the animal species and populations sampled and in the measurement of antimicrobial use and resistance. This variability prevented us from conducting a meta-analysis. These limitations highlight the need for standardized outcome measures or best practices for animal research to facilitate direct comparisons between studies and aid evidence synthesis across studies in the future. Future evaluations would also benefit from controlled study designs and longer follow-up, which could result in improvements in isolating the effects of policy and in assessing the sustainability of an intervention’s impact on antimicrobial resistance.

In conclusion, to address the challenge of antimicrobial resistance, governments and policy-makers must prioritize rigorous, transparent evaluations of policy interventions across all WHO regions, particularly in low- and middle-income countries, to strengthen the global evidence base and to inform effective One Health actions addressing antimicrobial resistance.
